# Effect of Physical Treatments on Functional Properties of Whey and Soy Protein Isolates in Oleogel Production Through Foam Template Method

**DOI:** 10.3390/molecules29225415

**Published:** 2024-11-16

**Authors:** Mojtaba Saneei, Sayed Amir Hossein Goli, Hajar Shekarchizadeh, Mehdi Rahimmalek, Antoni Szumny

**Affiliations:** 1 Department of Food Science and Technology, College of Agriculture, Isfahan University of Technology, Isfahan 84156-83111, Iran; m.saneei@ag.iut.ac.ir (M.S.); shekarchizadeh@iut.ac.ir (H.S.); 2 Department of Food Chemistry and Biocatalysis, Wrocław University of Environmental and Life Sciences, 50-375 Wroclaw, Poland; mrahimmalek@iut.ac.ir; 3 Department of Horticulture, College of Agriculture, Isfahan University of Technology, Isfahan 84156-83111, Iran

**Keywords:** soy protein isolate, whey protein isolate, foam, atmospheric cold plasma, ultrasonic, oleogel

## Abstract

This study aimed to investigate the impact of physical treatments, namely heat (70 °C for 48 h), atmospheric cold plasma (10 kW for 20 min), and ultrasonic frequency (40 kHz for 15 min), on the physicochemical and interfacial properties of soy protein isolate (SPI) and whey protein isolate (WPI) in the context of oleogel production by foam template (cryogel) method. The physical modification of both SPI and WPI was monitored using SDS-PAGE, Fourier transform infrared spectroscopy (FTIR) spectroscopy, and differential scanning calorimetry (DSC), measuring interfacial tension, color, solubility, foam volume, foam stability, and, finally, the density and oil absorption of the produced cryogel. The findings revealed that the application of ultrasonic waves resulted in a significant reduction in the content of alpha-helical of SPI and WPI while the other treatments increased the content of random coil proteins. FTIR analysis further showed that ultrasonic and heat treatment led to a decrease in C-N tensile vibration within the range of 1200–1650 cm^−1^ in SPI. Meanwhile, in cold plasma treatment, an increase was observed which was confirmed by the elevation of enthalpy from 100 to 128 kJ/kg. Physical treatments significantly altered the surface properties of both SPI and WPI, where this value was reduced in SPI and increased in WPI. The cold plasma method demonstrated superior performance in enhancing the solubility of SPI from 10 to 58.2%, while the solubility of WPI decreased from 96.4 to 90.4%. By modifying the proteins, the foam volume and oil adsorption ability of the related cryogel improved, as shown by the maximum oil absorption obtained after ultrasonic treatment for SPI (11.6 g/g) and cold plasma (9.17 g/g) for WPI. These results could be useful in applying physical treatments to modify proteins and create the cryogel as an oleogel template for structuring liquid oil and producing innovative health value-added foods.

## 1. Introduction

In recent years, people have shifted towards using unsaturated oils instead of saturated and trans fats, which have been linked to negative health impacts like cardiovascular diseases [1]. Oleogelation technology has gained a lot of attention for its ability to prepare semi-solid oils, known as oleogels, by embedding liquid vegetable oils in a three-dimensional gel network. Indirect methods such as the preparation of emulsion and foam [2,3,4] have been proposed to produce oleogels, while direct types are less preferred due to the ‘waxy’ texture and high processing temperature of the oleogel [3]. The foam-templated method is a newly emerging technique that uses specific polymers like proteins to prepare oleogels with desired properties [5,6]. This procedure entails the formation of foam utilizing biopolymers, which are subsequently subjected to freezing and drying processes to yield a cryogel, which is then saturated with oil by adding oil to the cryogel [3]. Finally, by shearing the saturated cryogel, oleogel is prepared. For the foam template assay, a foam with low density and enhanced mechanical properties as well as oil holding capacity is preferable, to produce an oleogel with desirable physical characteristics [7].

Proteins such as egg white [8], milk caseinate [9], soy protein isolate, whey protein [2], and rice bran protein [10], are commonly used in the foam template method due to their amphiphilic nature, availability, low cost, and health benefits [9]. Soy protein is extracted from soybeans, which are among the most abundant byproducts produced during the oil extraction process. It has an amino acid composition that is similar to the FAO/WHO recommendation [6]. Additionally, whey protein is also considered a high-quality protein with high nutritional value that is widely used in the food industry [11]. The physical and chemical properties of proteins can be modified to create foam templates, which can ultimately be used to form oleogels [12]. Improving protein ability in foam formation and stabilization can be achieved through chemical [13], enzymatic, and physical treatments [7]. Among these, the physical method is more favored due to the preference for natural products over those synthesized chemically or enzymatically [12,14]. Numerous studies have demonstrated the beneficial effects of various physical treatments, such as heat, dielectric barrier discharges of cold plasma [9,14], and ultrasonication [15], on the protein characteristics. In addition, heat treatment has been proven to enhance the foaming properties of proteins, and has gained popularity due to its simplicity and scalability. Heat has been reported to improve foam ability and its stability in whey and white egg protein due to the denaturation and unfolding of protein chains that enhance interfacial properties [8,16]. It has been discovered that gentle heat treatments (around 60 °C) can enhance the foaming properties of whey proteins, while stronger conditions (above 60 °C) can have a negative impact. The transformation of proteins from their natural state to the denatured state involves various changes in their secondary, tertiary, and quaternary structures, as well as constituent bond changes such as hydrogen bonding, hydrophobic interactions, electrostatic linkages, and disulfide bonds [17]. Cold atmospheric pressure plasma is a non-thermal technology that has a lot of potential in modifying and functionalizing protein molecular structures. One of the many advantages of cold plasma technology is its ability to modify proteins effectively at room temperature, making it ideal for heat-sensitive biopolymers. This technology produces ionized gases containing reactive oxygen and nitrogen species [9]. Previous studies have shown that exposing whey proteins and soy protein to mild oxidation by cold plasm (treatment less than 60 s (can enhance their forming capacity [18,19]. On the other hand, hard oxidation (cold plasma treatment for over 600 s) can lead to a decrease in the foamability of whey protein by causing protein aggregation [18]. Recent studies suggest that exposing whey protein to cold plasma for 10 s at 50 W can significantly reduce air–water interface tension and improve foam template preparation [19]. Researchers have also investigated the impact of cold plasma treatment on the structure and binding capacity of soy protein [19]. The results showed that the treatment increased β-sheets and random coil, while decreasing α-helix structure, ultimately leading to better foam formation [19]. Ultrasonic technology is another physical protein treatment that can be used to alter the properties of protein components in liquid media. This is achieved through a high-intensity, low-frequency ultrasound treatment, which generates mechanical acoustic waves that produce oscillating compression and rarefaction cycles in liquid media [20]. As a result of these compression cycles, small gas bubbles present in the liquid media grow in size and eventually reach a critical size, leading to their collapse, a process known as cavitation [15]. This can result in the formation of smaller and more uniform protein particles, thereby enhancing the foamability and stability of protein foams [21]. According to some research, the solubility and foam stability of barley protein isolate improved by approximately 20% after being subjected to ultrasonication for 30 min at 130 W [20]. In fact, sonication treatment could disrupt intermolecular bonds, leading to an increase in protein solubility [20]. Lastly, it was also reported that the foaming capacity of whey protein isolate was notably improved by ultrasound treatment at 20 kHz for 5 min. The ultrasonic treatment also increased the surface hydrophobicity of these proteins [21].

This study is the first to compare the effects of three physical treatments (heat, cold plasma, and ultrasonic) on two types of proteins to develop a foam-templated oleogel. Therefore, in this study, these three common physical treatments were performed on two different types of plant and animal proteins (SPI and WPI), with the aim of improving their physical and chemical properties. For this purpose, thermal behavior, molecular weight, functional groups, interfacial tendency, solubility, and color of each protein were assessed before and after the treatments. Subsequently, to approach the objectives of this study, the treated proteins were applied to fabricate foam, foam template (cryogel), and finally, oleogel. The knowledge obtained in this work could expand the potential of foam templates for the design of novel oleogels for numerous food applications.

## 2. Results and Discussion

### 2.1. Molecular Weight of Proteins 

**Figure 1:**
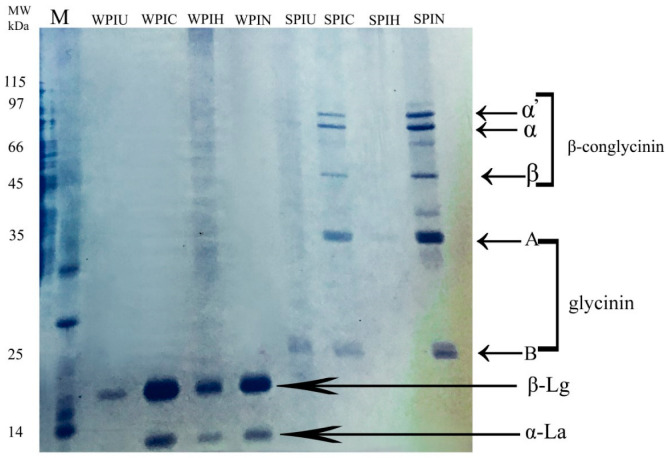
Visual appearance and SDS-PAGE patterns of SPI and WPI. Lane M: marker protein band. Lane MW: kDa molecular weight of marker. Lane WPIU: whey protein isolates ultrasonic treatment band. Lane WPIC: whey protein isolates cold plasma treatment band. WPIH whey protein heat treatment band. Lane WPIN: natural whey protein isolate. Lane SPIU: soy protein isolates ultrasonic treatment. Lane SPIC: soy protein isolates cold plasma treatment. Lane SPIH: soy protein isolate heat treatment. Lane SPIN: natural soy protein isolate. Soy protein isolates ά, α, and β subunits of β-conglycinin and the acidic and basic subunits of glycinin. Whey protein isolates α-lactalbumin (α-La) and β-lactoglobulin (β-Lg).

Sodium dodecyl sulfate-polyacrylamide gel electrophoresis (SDS-PAGE) is one of the most popular and important techniques for separating protein fractions based on molecular weight [22,23]. As can be observed in Figure 1, SPI 11s (glycine) consisted of two subunits: acidic (A) and alkaline (B), characterized by bands at ~34 kDa and ~20 kDa, respectively. Also, the 7S globulin (β-glycine), including the α, ά, and β subunits, was visible at bands of ~70–80 kDa and 58 kDa, respectively. In the WPI pattern, α-lactalbumin (α-La) appeared at ~14 kDa, and β-lactoglobulin (β-Lg) was observed at ~18 kDa. The obtained results from SDS-PAGE analysis of SPI and WPI were similar to previous reports on their molecular weights [24,25]. The bands for native SPI were observed between 20 and 80 kDa. However, both ultrasonication and heating treatments led to the denaturation of SPI, as evidenced by the SPIU and SPIH electrophoretic patterns. 

However, after heat and ultrasound treatment, the main protein bands of SPI became lighter in color, which means that the protein contents at this molecular weight were reduced. The disappearance of SPIH bands might be attributed to denaturation or decomposition due to heating. This could result in the formation of either complex or smaller units that may either leave quickly or accumulate within the gel column [24,25]. Ultrasonication treatment directly increased the frequency and affected all external bonds in the protein, as SPIU lost almost all protein monomers. Previous work showed that ultrasonication treatment at 20 kHz causes a significant decrease in β-glycine activity. Researchers also reported their changes in the molecular weight profiles of pumpkin seed protein after ultrasonic treatment, indicating that the ultrasound treatment changed the protein isolates’ primary structure [7].

It was noted that the electrophoretic profiles of SPI and WPI samples did not exhibit significant differences when compared to those of the treated SPIC and WPIC samples. Moreover, the analysis revealed no formation of either covalent bonds or disulfide (S–S) bonds between the protein molecules [19].

In Figure 1, it can be observed that the β-Lg monomer band in WPIN had a more noticeable color compared to WPIH, which could be attributed to the increased formation of internal protein bonds. The distinct α-La band disappeared after the heat treatment, while the β-Lg bands shifted to a higher molecular weight, indicating their interaction. In the case of WPIU, the band faded, resulting from the partial denaturation of the protein and the creation of peptides due to the treatment.

### 2.2. Identification of Functional Groups

**Figure 2:**
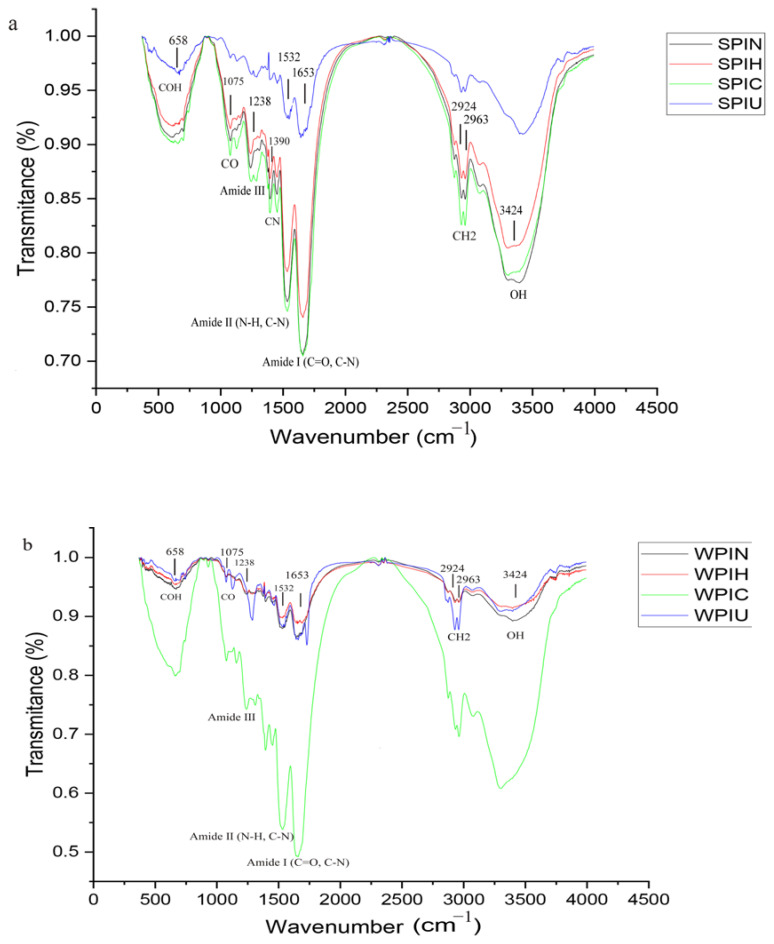
Fourier transform infrared spectroscopy spectra of SPIN (natural soy protein isolate), SPIH (heat-treated soybean protein isolate), SPIC (soybean protein isolate treated with cold plasma), and SPIU (ultrasonic-treated soybean protein isolate) (**a**). Infrared conversion spectroscopy of Fourier transform of WPIN (natural whey protein isolate), WPIH (heat-treated whey protein isolate), WPIC (cold plasma-treated whey protein isolate), and WPIU (ultrasonic-treated whey protein isolate) (**b**).

A reliable method for analyzing protein structure is FTIR, which can predict a protein’s secondary structure based on its peptide group arrangement [26]. Figure 2a,b exhibit the FTIR spectrum of the proteins with the fingerprint region. The fingerprint region from 1000 to 1700 cm^−1^ is important, showcasing various variations in biological samples, particularly amides. Amide vibrations are included as follows: The two main bands of the protein infrared spectrum are amide I and amide II. The amide I band (between 1600 and 1700 cm^−1^) is mainly associated with the C=O stretching vibration. Amide II results from the N-H bending vibration and the C-N stretching vibration. Amide III is a very complex band, resulting from a mixture of several coordinate displacements. Also, the bands corresponding to the secondary structure were as follows: α-helix: 1648–1657 cm^−1^, β-sheet: 1623–1641 cm^−1^ and 1670–1690 cm^−1^, β-turn: 1660–1670 cm^−1^ and 1690–1700 cm^−1^, random coils: 1637–1645 cm^−1^ [19].

The bands at 658 and 1075 cm^−1^ wavenumbers indicated the COH and C-O groups in the protein. During the ultrasonic treatment, the intensity of the bands at 658 and 1075 cm^−1^ decreased, while they intensified during cold plasma treatment. This region also showed a slight shift, indicating minor degeneration due to the treatment. The bands located at around 1653 cm^−1^ are associated with the vibration of C=O, which is the primary template in amide Ⅰ. The band at 1532 cm^−1^ represents the stretching of the N-H, known as amide II. However, as depicted in Figure 2a, the intensity of the dianionic symmetric stretching in both amid Ⅰ and amide II bands decreased due to the ultrasonic and thermal treatments. This reduction demonstrates the degradation of functional groups within SPI and indicates that denaturation occurred as a result of the treatment. A similar report has been published, indicating that temperatures exceeding 80 °C during treatments decreased the intensity of bands associated with the random coil in the 1637–1645 cm^−1^ region [16]. Additionally, subjecting protein to ultrasonic treatment at 16 kV for 5 min enhanced the structures of amide Ⅰ, amide Ⅱ, and amide Ⅲ [19]. Based on our observations, the cold plasma treatment resulted in a decrease in the α-helix structure (1648–1657 cm^−1^) of SPI but an increase in the β-sheet (1623–1641 cm^−1^). Additionally, the 2000 to 2200 cm^−1^ region is linked to protein crystallization adsorption, with a distinct pattern observed in ultrasonic treatment. The band at 2924 to 2963 cm^−1^ also indicates CH_2_ groups, but no significant difference was observed among the samples in this region. The band observed in the 3400 cm^−1^ region corresponds to the OH bond in SPI samples. This band is reduced in all types of treatments, especially during ultrasonic treatment. This reduction is likely due to a decrease in the bonding levels of the OH groups and a lower moisture content.

The functional groups of WPI protein are illustrated in Figure 2b. The proportion of amide I to amide II was noticeably higher in SPIN compared to that in WPIN, suggesting that there were dissimilarities in the protein conformation pattern between these two samples. There were slight variations in the structure of WPI by applying the treatments. It can be determined that α-La and β-La were present based on the spectral range of 1000 to 1600 cm^−1^. The band at approximately 1070 cm^−1^ indicated the presence of lactose monohydrate, which disappeared after being subjected to heat treatment (confirmed by SDS-PAGE, as illustrated in Figure 1) likely due to the Millard browning reaction. The band around 1238 cm^−1^ showed amide Ⅲ, which was removed during heat treatment. The previous study showed heat treatment at 80 °C for 600 s could denature WPI. The helical structure was reduced and exposed to more hydrophobic groups [13,27]. The presence of bands ranging from 1510 to 1540 cm^−1^ was an indication of amide Ⅱ in the structure. The large band observed at 1530 cm^−1^, as shown in Figure 2b, was linked to the configuration of amide Ⅱ that is part of the N-H and C-N groups. The results of this study were consistent with research that identified the 1533 cm^−1^ band as a mixture of N-H and C-N [21]. Furthermore, the study found that treatment with cold plasma resulted in the removal of amide Ⅱ, CO, and NH groups in the 1517 cm^−1^ range [27].

The two-dimensional structure of WPIN, in the form of amide Ⅰ, can be evaluated in the range of 1600 to 1700 cm^−1^, which includes various types of β-turn and β-sheet structures, random coil, and α-helix. The region of 1640 to 1650 cm^−1^ (amide Ⅰ) specifically highlights the presence of random coils, as indicated by the band at 1640 cm^−1^ in the spectrum. Cold plasma treatment was found to be more effective, possibly due to the present differences in the preservation of functional groups in the protein. After treatment with cold plasma, the sample displayed a rise in α-helix and aggregated β-sheet structure, along with a decrease in the random coil, β-sheet, and β-turn. These findings were consistent with protein solubility and the protein grouping strip in the gel electrophoresis test (Figure 1) [19].

The subsequent range of 1660 to 1700 cm^−1^ is associated with the β-turn. The results for WPIC exhibited a significant divergence from other treatments within this range, which may be attributed to the formation of two-dimensional structures. The intensity of the CH_3_-R band can be interpreted as an indication of the presence of moisture and fat [28]. Four different spectra showed the effect of applied treatments on the structures and moisture content of WPI. The zone from 2800 to 3000 cm^−1^ is recognized as oil and fat [24]. Wavenumbers at 2924 and 2855 cm^−1^ belonged to the (-CH_2_) and (-CH_3_) groups of milk fatty acid. The recent band (2855 cm^−1^) was not visible in the protein treated with ultrasonic waves and cold plasma, which might be due to the transition from saturated to unsaturated fatty acids. The region between 3600–3100 cm^−1^ is identified as the amide I region, which exhibits a broader peak with the main intensity at around 3300 cm^−1^ (as shown in Figure 2b). This peak is associated with the stretching vibration of the hydroxyl (OH) group. Following the cold plasma treatment, a notable increase in absorption was observed in this region. This phenomenon can be attributed to the enhancement of polar groups on the surface of the protein. [28].

### 2.3. Thermal Behavior

**Figure 3:**
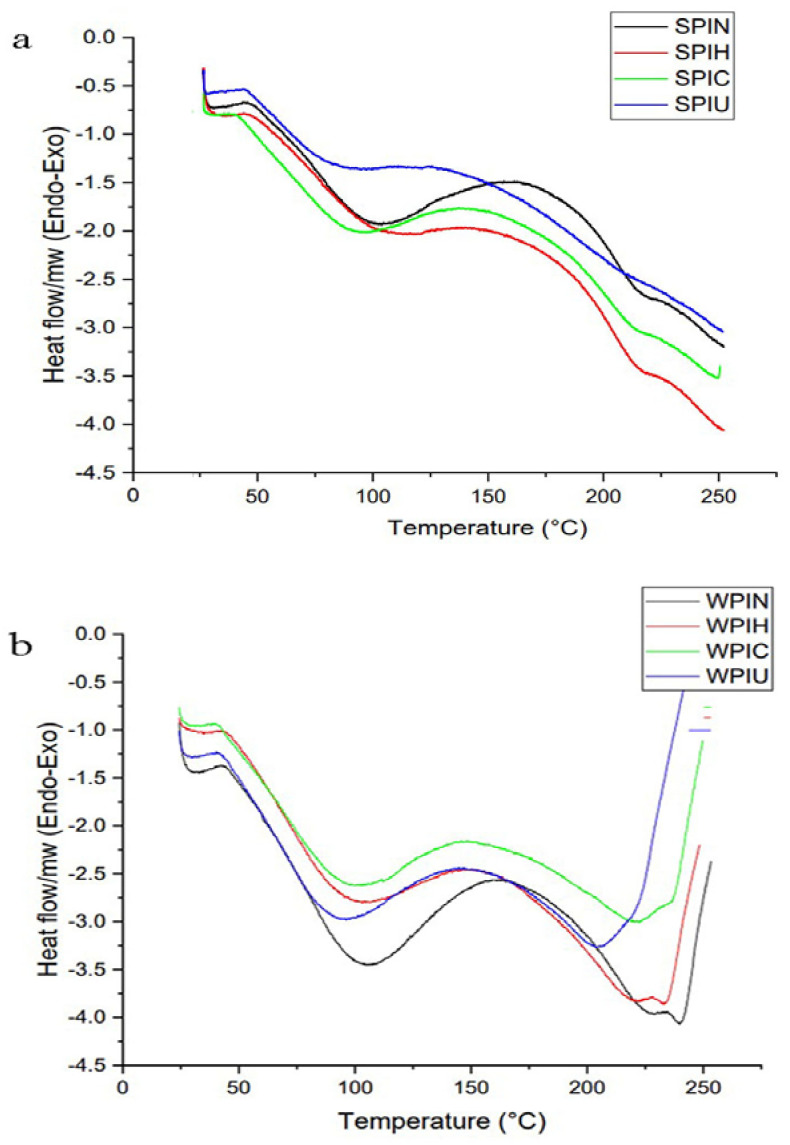
DSC thermograms of SPI (**a**) and WPI (**b**).

The DSC thermograms for SPI and the treated protein are illustrated in Figure 3a. The two peaks shown in the SPI diagram likely corresponded to the two main protein components present in the protein, namely the 7S (75.46 °C) and the 11S (123.51 °C) fractions, as shown in Table 1 and Figure 3a. The higher the denaturation temperature (7S fraction), the higher the energy will be required to denature the protein. A comparison of different treatments showed that the enthalpy increased in SPIC by 28% but a decrease in enthalpy by more than 50 and 10% could be observed in the heat and ultrasonic treatments, respectively. Also, protein melting temperature showed a reducing trend as cold plasma processing. Based on the result of SDS-PAGE, it could be confirmed that heating has denatured the secondary structure of SPI (Figure 1). The β-conglycinin segment within the gel profile of SPIH did not show this region. The enthalpy of SPI could be decreased by heat treatment at any temperature and duration [23]. A reduction in denaturation temperature from 77.10 °C (untreated) to 65.90 °C (125 V Plasma) was observed for zein protein [29].

The presence of two peaks in Figure 3b, with an endothermic peak at 100 °C, indicated the initial composition of β-la and α-la in WPI. The first peak was associated with β-la monomer, which is the main component of natural whey protein isolate (WPIN). After the application of heat treatment, there was a reduction in enthalpy of approximately 40%. Similar effects were observed on SDS gel, where bands associated with β-la and α-la, having molecular weights of 18.36 kDa, and 14.17 kDa, respectively, decreased after heat treatment. According to another research, high temperatures could lead to the degeneration of WPI, resulting in the destruction of the network and a decrease in enthalpy [30]. The enthalpy values could be used to analyze the structural changes that occurred in the WPI during ultrasonic treatment. Following the ultrasonic process, there was a decrease in enthalpy by 63% and a decrease in the peak temperature to 93 °C. Ultrasound treatment disrupted the interconnected WPI network and broke hydrogen bonds, leading to lower enthalpy. The impact of ultrasonic treatment on enthalpy was less than that of heat treatment because the effect of heat on molecular bonds was more significant than that of ultrasonic treatment [15].

It appeared that cold plasma treatment had a minimal impact on enthalpy, with a decrease in the peak’s initial temperature. Therefore, the effectiveness of cold plasma treatment in either forming or breaking bonds within WPI was limited. Based on our results, cold plasma modification has been confirmed by spectroscopy to increase the quantity of amide Ⅰ and Ⅱ. These structures were presented in the α-helix, β-sheet, and random coil sections of β-la and α-la. High-energy bonds like C-O and C-N were replaced with lower energy bonds, such as C-OH [4].

### 2.4. Surface Tension

**Figure 4:**
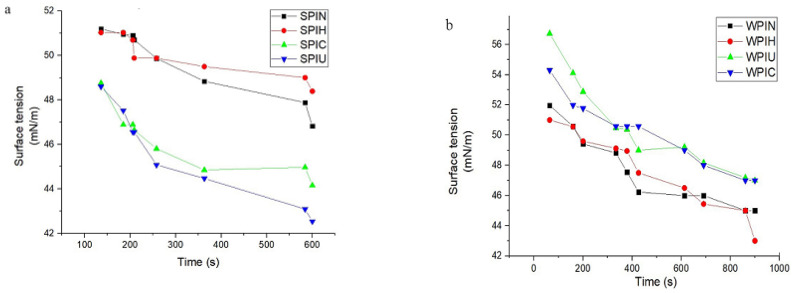
Surface characteristics of SPIN (natural soy protein isolate), SPIH (heat-treated soy protein isolate), SPIC (atmospheric cold plasma-treated soy protein isolate), and SPIU (ultrasonic-treated soy protein isolate) (**a**). Surface characteristics of WPIN (natural whey protein isolate), WPIH (heat-treated whey protein isolate), WPIC (atmospheric cold plasma-treated whey protein isolate), and WPIU (ultrasonic-treated whey protein isolate) (**b**). The concentration of SPI and WPI is 0.001 wt/volume.

The surface tension of protein systems heavily relies on the combination and concentration of surface-active agents present. The rate of diffusion of surfactants towards the surface determines the composition of the monomolecular layer at the interface. Additionally, surfactants adsorbed at the interface can lead to protein unfolding and denaturing. Replacement of original surfactants with more surface-active agents can also occur [7]. Figure 4a,b show the changes in surface tension of SPI and WPI at the air–water interface over time. The observation conditions remained constant in all treatments for 600 and 800 s. The surface tension of both SPIN and SPIH started at 51 mN/m and gradually decreased to 50 mN/m after 250 s. After approximately 350 s, SPIN reached a surface tension of 49 mN/m, decreasing faster than SPIH. The surface tension of both SPIN and SPIH steadily decreased over 550 s and reached 47 ± 0.8 and 48 ± 0.8 mN/m, respectively, at the 600 s mark. The surface activity of SPIC and SPIU was higher than that of SPIN and SPIN, which indicated their better performance as emulsifiers. The presence of a hydrophobic group played an important role in the adsorption ability of SPIU and SPIC, confirming previous results that reported that the minimal surface activity of SPI was about 50 mN/m. Various treatments have been applied, causing surface tension changes due to functional groups [6].

WPIN initially had a higher surface tension than SPIN, which could be attributed to WPIN peptides’ higher presence in the air bubble’s surface area. As seen in Figure 2, surface active agents unequivocally caused escalated surface activity of WPI, which suggests that WPI was adsorbed at the interface of air and water. As indicated in all treatments, there was an increase in the initial amount of surface tension that may be due to protein denaturation, which causes a decrease in solubility and an increase in hydrophobicity. It was also documented that physical treatment causes reorganization of the structure of WPI molecules, which in turn causes an increase in protein diffusion [15]. After 500 s, there was a significant drop in WPIC levels compared to the other treatments. WPIC and WPIU displayed a similar but more pronounced decrease relative to SPIN. This could be attributed to the solubility difference between the two proteins. The surface tension of whey protein was around 55 mN/m, consistent with previous findings. Additionally, ultrasonic treatment resulted in a significant reduction in interfacial tension, as observed in previous studies [31].

### 2.5. Characterization of Cryogel and Oleogel 

#### 2.5.1. Color

The influence of various treatments on the physicochemical properties of both proteins is shown in Table 2. The differences in L* values between SPIU and the other variants—SPIC, SPIN, and SPIH—were noticeable. The L* value exceeded 3, falling within the range of 78 to 82, which was attributed to the ultrasonic treatment that influenced the darkening of SPIU (see Figure 2a). Additionally, the a* value increased in both SPIH and SPIU due to their higher rates of brown pigment formation at elevated temperatures [13,26]. However, there were no significant differences between the treatments in the b* value. WPIH changed the L*, a*, and b* values from 89.3 to 78.6, 0.03 to 8.4, and 15.9 to 37, respectively. This indicated that the samples became darker and yellower with heat treatment, as evidenced by increases in the b* values (where negative numbers represent blueness and positive numbers represent yellowness). After 48 h with treatment of WPIH, the a* value increased to approximately 200 times higher than that of untreated control samples. These changes indicated the reactive formation of the pigmented end product. Heat treatment may have caused the formation of a complex between α-la and β-Lg, and led to an increase in darkness [32]. The hydrolysis of polypeptide to monopeptide increased the number of reactive groups available for non-enzymatic browning reactions [33].

#### 2.5.2. Solubility

As shown in Table 2, all applied treatments increased the solubility of soy protein, Although the solubility of SPI was not as high as WPI, as indicated in Figure 2a, the increase may be attributed to the absence of connections between hydrophobic groups. The increase in solubility could be attributed to the denaturation of SPI, leading to a rise in the particle–liquid phase compared to that in the particle–particle phase. Our findings aligned with another research suggesting that physical treatment might affect solubility by altering proteins and exposing hydrophilic amino acid residues [34,35]. The solubility of WPIN and WPIU dispersions was significantly higher than that of WPIC and WPIH dispersions, with a percentage of around 95% and 96%, respectively. It aligned with previous research suggesting that cold plasma treatments could decrease the solubility of animal proteins like WPI by forming insoluble protein aggregates and increasing particle size [9]. Moreover, heat treatment of WPI above their denaturation temperatures can also decrease solubility [16].

#### 2.5.3. Foam Volume and Stability

The volume and stability of foam are essential parameters that impact the resulting cryogels in the foam-templated method, since the physical properties of aqueous foams directly influence the network structure of the cryogels [5]. The foaming volume (FV) and foam stability (FS) of SPI and WPI in different treatments were determined, as shown in Table 2. FV of SPIH, SPIC, and SPIU significantly increased by 4, 5 and 3.7 times, respectively. The highest FV reached ~500% belonging to SPIC. It can be argued that the functional properties of SPI, particularly the FV, have been impacted by physical treatments, especially cold plasma treatment. It appears that the discrepancies stem from structure variations, as outlined in Figure 1 and Figure 3. This aligns with previous research that identified cold plasma as a significant factor that impacts the foaming ability and functional traits of rice bran protein [10]. Unlike SPIN, WPIN had the highest FV, and all treatments led to the breakdown of the foam’s network structure. Heat treatment reduces the foaming properties of whey protein, likely because protein aggregation is associated with a decrease in the solubility of WPI [36].

Cold plasma almost increased the solubility of SPI by about six times while having the opposite effect on FS. Among all the treatments applied for SPIN, only ultrasonic treatment did not cause any change in FS. However, both cold plasma and heat treatments decreased resistance, as noted in Table 2. The treatments that increased solubility could lead to a decrease in stability. The stability of air bubbles in WPI depended on its surface properties. WPIN produced approximately twice the foam volume of WPIC while maintaining equal foam stability. SPIN foam was much more stable (around 30 times) than WPIN, which might be due to the higher solubility of WPI compared to SPI. The differences between SPI and WPI in terms of solubility, FS, and FV could be fully understood by examining various strips of acrylamide gel, as shown in Figure 1. Based on Table 2, smaller protein molecules resulted in lower foam stability, while higher molecular weight monomers in SPI led to more stable foam. All treatments decreased FV of WPI and the lowest FV was found for WPIC (480%), which was close to the SPIC (500%). Our findings align with previous research that suggested that the size of SPI may cause molecular aggregation, leading to a thicker interface layer at the air/water interface. This thicker layer could potentially increase the FS [19].

#### 2.5.4. Cryogel Density

The production of the temple with high oil absorption efficiency is related to the density of cryogels. The density of cryogels ranged from 0.03 to 0.01 g/m. Previous studies have demonstrated that the density of commercial porous materials, such as WPI foam (known as temple), is 0.01 g/mL [9]. The density increased threefold in SPIH due to the agglomeration of polypeptides, while ultrasound and cold plasma did not significantly change the density of the SPI density (*p <* 0.05). The impact of treatments on WPI was insignificant as there were no noticeable structural changes that could affect the density of cryogel in a dry state.

#### 2.5.5. Oil Adsorption

Cryogel capability for oil adsorption is one of the essential physicochemical factors for evaluating cryogel structure. Notably, the highest oil absorption (11.68 g/g) was found in SPIU, followed by SPIC (9.41 g/g). The oil adsorption of SPIH was less than 5 g, which was attributed to the higher density and more compact network of the cryogel. On the other hand, the oil adsorption values of WPI ranged from 4.35 to 9.17 g/g. The oleogel that was prepared with WPIU had the lowest value. The ultrasonic intensity was so high that it destroyed one of the protein fractions involved in the structure, reducing oil absorption in the cryogel structure. These results also supported the trends observed in the SDS gel data (Figure 1). In contrast, the sample treated with cold plasma showed the highest value. The compression of the template structure of the oleogel formed by WPIC may be the only reason. Furthermore, the protein template has been reported to have an impact oil absorption and oil binding capacity [2].

## 3. Materials and Methods

### 3.1. Materials

The soy protein isolate (SPI) containing 83% protein and the whey protein isolate (WPI) containing 92% protein were purchased from Karen Co (Tehran, Iran). Canola oil was obtained from a local supermarket. All analytical-grade chemicals were sourced from Merck Co. or Sigma-Aldrich (Steinheim, Germany).

### 3.2. Protein Treatment

#### 3.2.1. Heat Treatment 

A certain amount of protein (15–20 g) was heated in the oven at 70 °C for 48 h and then allowed to cool to room temperature (20 to 25 °C) [36].

#### 3.2.2. Dielectric Barrier Discharges Cold Plasma Treatment

Three grams of protein, each measuring 1 mm in thickness, were placed on a plate. The samples were then subjected to a 20 min electric discharge at 10 kW using a dielectric barrier discharge plasma generator (Kavosh Yaran Fan Pooya Co., Tehran, Iran) under standard atmospheric conditions. The distance between the electric discharge plate and the sample was 0.5 mm [19].

#### 3.2.3. Sonication Treatment

The protein was first dissolved in water at a concentration of 2% *w*/*v* and kept at room temperature for 24 h to maximize water absorption. The 100 mL solution was stirred for 10 min, then transferred to a 250 mL beaker and placed in an ice bath. Then, the solution was treated with ultrasonic power of 130 W and 40 kHz for 15 min (Farasout, Tehran, Iran), using a 13 mm diameter titanium probe placed 1 cm from the bottom of the treatment cylinder. Finally, the solution was dried using a freeze dryer (Alpha 1–2 LD plus, Schönwalde-Glien, Germany) [15].

### 3.3. Characterization of Treated Protein

#### 3.3.1. Polyacrylamide Gel Electrophoresis (SDS-PAGE)

The molecular weight was analyzed using sodium dodecyl sulfate-polyacrylamide gel electrophoresis (SDS-PAGE) on a Mini-PROTEAN Tetra Cell (Bio-Rad Laboratories, Hercules CA, USA). Protein powder samples were mixed with 5× sample buffer, containing the following components: 0.5M Tris-HCl (pH 6.8), 20% glycerol (*v*/*v*), 1% SDS (*w*/*v*) 0.05% bromophenol blue (*w*/*v*), and 0.74% β-mercaptoethanol (*w*/*v*). The mixture was then heated at 100 °C for 5 min. Next, the samples were loaded onto gels, where a 12% separating gel and 4% stacking gel were used. The loaded gels were stained with Coomassie Blue R-250 (Sigma) at room temperature. Finally, the separated protein bands were identified by comparing them with standard protein markers from SMOBIO Technology Inc. in Hsinchu, Taiwan. The standard protein marker, also known as molecular weight marker, displays a range of molecular weights from 10 to 180 kDa. The gel was then scanned and the images analyzed with Image J2 software.

#### 3.3.2. Fourier Transform Infrared Spectroscopy (FTIR)

The SPI and WPI powders were mixed with potassium bromide and analyzed using a PerkinElmer FTIR spectrometer (Perkin Elmer Co., Shelton, Connecticut, USA). The Fourier transform infrared (FTIR) analysis was conducted at a resolution of 4 cm^−1^ across a spectral range of 400 to 4000 cm^−1^. Subsequently, the FTIR spectra were processed using OriginPro 2017 software to evaluate functional groups [11].

#### 3.3.3. Differential Scanning Calorimetry (DSC)

The thermal properties of both raw and treated SPIs and WPIs were investigated using a DSC (DSC 250, TA Instruments, Newcastle, DE, USA). The proteins were scanned over a temperature range of 25 to 250 °C at a rate of 5 °C per minute. Approximately 5 mg of each powder was placed in hermetically sealed aluminum pans under an inert atmosphere with an empty pan used as a reference [9,34]. The data were analyzed using TA Universal Analysis software 4.5 A.

#### 3.3.4. Protein Solubility

First, a dilution of each protein (1:10 *w*/*v*) was prepared and poured into a 15 mL centrifuge tube and centrifuged at 44274 rcf for 15 min. Subsequently, the upper phase of the solution was separated, and its absorbance was determined using a UV-Visible/NIR Spectrophotometer (UH5700 Minato-ku, Tokyo, Japan) at 562 nm. Solubility was calculated based on the total solids content of the upper phase of the solution expressed as a percentage of the total solids content of the samples prior to centrifugation [34].

#### 3.3.5. Color Parameters 

The color parameters of the SPI and WPI powders were evaluated based on the values of L* (lightness), a* (redness), and b* (yellowness) values using the ZE-6000 color meter (NIPPON DENSHOKU, Osaka, Japan) [13,35].

#### 3.3.6. Interfacial Tension

The interfacial tension of air bubbles was determined using an automat drop tensiometer (Apex Technolog.Co., Arak, Iran). Interfacial drop tension was measured through the drop shape analysis of air bubbles in a solution containing 0.01% (*w*/*v*) protein. A bubble (10–40 μL) was formed at the tip of a needle, and its shape was captured with a CCD camera under a uniform light source until the bubble disappeared [4].

### 3.4. Foam, Cryogel, and Oleogel Preparation

**Figure 5:**
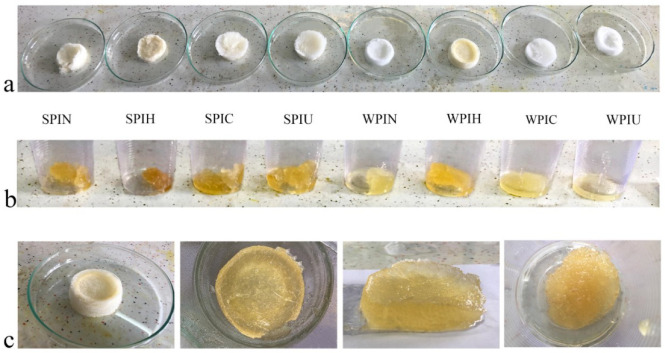
Cryogel and oleogel images resulting from various protein treatments. (**a**) Cryogels produced from natural and treated protein by foam method. (**b**) Final oleogel produced from cryogel. (**c**) Production steps of oleogel from cryogel include oil absorption and homogenization. SPIN: natural soy protein isolate. SPIH: heat-treated soy protein isolate. SPIC: atmospheric cold plasma-treated soy protein isolate. SPIU: ultrasonic-treated soy protein isolate. WPIN: natural whey protein isolate. WPIH: heat-treated whey protein isolate. WPIC: atmospheric cold plasma-treated whey protein isolate. WPIU: ultrasonic-treated whey protein isolate.

To prepare the foam, approximately 3 g of protein was added to distilled water and mixed using a mixer at 20 °C for 15 min. The resulting solution was then adjusted to a volume of 200 mL with distilled water. Next, the solution was aerated at 13,000 rpm using an IKA T 25 Digital Ultra-Turrax homogenizer for 5 min. To produce cryogel, the foams were transferred to an aluminum tray (measuring 2 cm × 5 cm) and immediately frozen at a temperature of −76 °C for 24 h. Thereafter, the frozen foams were dried for 72 h using a freeze dryer (Alpha 1–2 LD plus, Schönwalde-Glien, Germany). To prepare the oleogels, canola oil was added to the cryogel until it was fully saturated with edible oil. The mixture was allowed to rest for 1 h without shearing to allow any excess oil to be removed (Figure 5) [12].

### 3.5. Characterization of Hydrogel, Cryogel, and Oleogel

#### 3.5.1. Foam Volume

A reported method with some modifications, was used to measure the foam volume (FV) [8]. A standard 50 mL cylinder was filled with foam at room temperature. After converting the foam into a solution by heating, the measurement was taken based on the difference between the volumes of the dispersion and the foam, termed as foam volume [37].

Equation (1):

(1) $$\;\%\;FV=\frac{\;\left(V\;foam\right)-\left(V\;solution\right)}{\left(V\;solution\right)}\times 100$$

#### 3.5.2. Foam Stability 

Foam stability (FS) was measured by the time required to lose 50% of the foam volume [37].

#### 3.5.3. Apparent Density of Cryogel

The density of cryogels can be calculated using Equation (2) after measuring the radius and height of a lyophilized cryogel. In this equation, ‘mL’ refers to the weight of the lyophilized cryogel, and ‘vL’ represents the volume of the cylinder-shaped lyophilized cryogel [11].

Equation (2):

*d* = *m_L_*/*v_L_* (2)

#### 3.5.4. Oil Absorption of Cryogel

The transformation of cryogel into oleogel involves the absorption of edible oil within its pores. Following the method described by Manzocco et al. (2017) with slight adjustments, the cryogel samples were immersed in 250 mL beakers containing 125 mL of edible oil, allowing the pores of the cryogel to be saturated with the oil. Excess oil was removed by placing the saturated cryogel on absorbing paper for 30 min, after which the cryogel is weighed. The amount of absorbed oil was determined by calculating the difference between the weight of cryogel before and after oil absorption. Subsequently, the oil-containing sample underwent thorough mixing at 10,000 rpm using an IKA T 25 Digital Ultra-Turrax homogenizer for approximately 2–5 min to achieve oleogel with a consistent texture [5].

### 3.6. Statistical Analysis

The reported values represent the mean of three separate replicates and were analyzed using a one-way analysis of variance (ANOVA). Significant differences (*p* < 0.05) among the treatments were determined using the least significant difference (LSD) test.

## 4. Conclusions

This research delved in the impact of heat, ultrasonic waves, and cold plasma on the functional properties of SPI and WPI to optimize the production of cryogel for implementation in oleogel production. Ultrasonic treatment induced a notable shift in the FTIR spectrum, resulting in a reduction of secondary structure. Cold plasma treatment exhibited minimal influence on random coils and α-helices, while heat treatment led to a modest alteration. These treatments demonstrated an enhancement in SPI’s solubility and foam volume, while decreasing those parameters in WPI. Notably, ultrasonic treatment of SPI and cold plasma treatment of WPI facilitated the creation of cryogels with high oil absorption. This work showed that the modification of proteins, as the main backbone of cryogels, could be a method to improve the physicochemical and rheological properties of the oleogel produced through the foam template method, although more studies are necessary to confirm this hypothesis.

## Figures and Tables

**Table 1:** The main parameters in heating behavior of various proteins measured by differential scanning calorimetry.

Treatments	Enthalpy (J/g)	Maximum Peak (°C)	Offset (°C)	Onset (°C)	Peak Numbers
SPIN	100.03 ± 1.2 ^b^	98.37± 6.4 ^a^	123.51 ± 4.8 ^b^	75.46 ± 1.1 ^a^	2
SPIH	42.25 ± 3.3 ^c^	97.10 ± 4.7 ^a^	119.34± 2.2 ^b^	75.50 ± 2.6 ^a^	2
SPIC	128.69 ± 1.9 ^a^	91.63 ± 2.9 ^b^	119.08 ± 6.1 ^b^	51.37 ± 1.4 ^c^	2
SPIU	94.39 ± 0.6 ^b^	78.53 ± 3.3 ^c^	111.63 ± 5.4 ^c^	64.71 ± 2.8 ^b^	2
WPIN	155.58 ± 3.0 ^a^	100.56 ± 4.5 ^a^	129.89 ± 0.8 ^a^	66.12 ± 0.9 ^b^	2
WPIH	59.58 ± 5.4 ^c^	95.50 ± 2.0 ^b^	126.65 ± 1.9 ^b^	64.43 ± 5.7 ^b^	2
WPIC	100.91 ± 0.8 ^b^	95.16 ± 0.4 ^b^	112.60 ± 1.0 ^c^	44.97 ± 1.2 ^c^	2
WPIU	57.54 ± 4.9 ^c^	93.00 ± 0.6 ^b^	120.21 ± 3.2 ^b^	63.68 ± 0.6 ^b^	2

SPIN: natural soy protein isolate. SPIH: heat-treated soy protein isolate. SPIC: cold plasma-treated soy protein isolate. SPIU: ultrasound-treated soy protein isolate. WPIN: natural whey protein isolate. WPIH: heat-treated whey protein isolate. WPIC: cold plasma-treated whey protein isolate. WPIU: ultrasound-treated whey protein isolate. The values with different lowercase letters (a, b, c, d) within the same column are significantly different (*p* < 0.05).

**Table 2:** Effect of physical treatments on physicochemical properties of SPI and WPI.

Samples	Color			Solubility (%)	Foam Volume (%)	Foam Stability (min)	Oil Adsorption (g/g)	Density (g/mL)
	L*	a*	b*					
SPIN	82.8 ± 4.5 ^a^	2.8 ± 1.4 ^b^	18.5 ± 3.3 ^ab^	10.0 ± 2.2 ^c^	101 ± 1.4 ^c^	145 ± 1.0 ^a^	7.59 ± 4.1 ^b^	0.018 ± 0.0 ^a^
SPIH	82.0 ± 0.0 ^a^	3.3 ± 5.6 ^a^	21.6 ±6.7 ^ab^	21.0 ± 3.0 ^b^	400 ± 0.0 ^ab^	128 ± 2.7 ^b^	4.19 ± 0.1 ^c^	0.030 ± 0.6 ^b^
SPIC	82.9 ± 0.7 ^a^	2.5 ± 2.7 ^b^	18.4 ± 6.0 ^ab^	58.2 ± 2.9 ^a^	500 ± 1.2 ^a^	120 ± 6.5 ^b^	9.41 ± 1.2 ^ab^	0.010 ± 4.5 ^a^
SPIU	78.4 ± 9.0 ^b^	3.4 ± 3.2 ^a^	20.3 ± 3.6 ^ab^	38.8 ± 6.6 ^ab^	370 ± 1.9 ^b^	157 ± 3.3 ^a^	11.68 ± 2.9 ^a^	0.010 ± 0.0 ^a^
WPIN	89.3 ± 1.2 ^a^	0.03 ± 6.3 ^c^	15.9 ± 7.4 ^b^	95.4 ± 8.1 ^a^	790 ± 18.1 ^a^	5 ± 2.7 ^b^	7.09 ± 0.1 ^b^	0.010 ± 1.3 ^b^
WPIH	78.6 ± 5.3 ^b^	8.4 ± 5.6 ^a^	37.0 ± 3.0 ^a^	90.8 ± 1.7 ^b^	500 ± 20.5 ^b^	8 ± 7.0 ^ab^	8.97 ± 3.4 ^a^	0.015 ± 4.6 ^a^
WPIC	87.8 ± 4.0 ^a^	1.5 ± 4.0 ^b^	18.2 ± 8.3 ^b^	90.4 ± 3.7 ^b^	480 ± 1.2 ^b^	5 ± 3.0 ^b^	9.17 ± 2.0 ^a^	0.016 ± 0.2 ^a^
WPIU	85.6 ± 0.1 ^ab^	0.13 ± 0.0 ^ab^	11.7 ± 0.2 ^c^	96.4± 0.2 ^a^	590 ± 3.6 ^a^	12 ± 0.2 ^a^	4.35 ± 0.0 ^c^	0.010 ± 0.0 ^b^

SPIN: natural soy protein isolate. SPIH: heat-treated soy protein isolate. SPIC: atmospheric cold plasma-treated soy protein isolate. SPIU: ultrasonic-treated soy protein isolate. WPIN: natural whey protein isolate. WPIH: heat-treated whey protein isolate. WPIC: atmospheric cold plasma-treated whey protein isolate. WPIU: ultrasonic-treated whey protein isolate. All data were measured with an average of three replications. The values with different lowercase letters (a, b, c, d) within the same column are significantly different (*p* < 0.05).

## Data Availability

Data is contained within the article.
